# The application of eDNA for monitoring of the Great Crested Newt in the UK

**DOI:** 10.1002/ece3.1272

**Published:** 2014-09-30

**Authors:** Helen C Rees, Keith Bishop, David J Middleditch, James R M Patmore, Ben C Maddison, Kevin C Gough

**Affiliations:** 1 ADAS UK Ltd, School of Veterinary Medicine and Science, The University of NottinghamSutton Bonington Campus, Leicestershire, LE12 5RD, U.K; 2 School of Veterinary Medicine and Science, The University of NottinghamSutton Bonington Campus, Leicestershire, LE12 5RD, U.K; 3 ADAS Boxworth, ADAS UK LtdBoxworth, Cambridgeshire, CB23 4NN, U.K; 4 ADAS Wolverhampton, ADAS UK LtdPendeford House, Pendeford Business Park, Wobaston Road, Wolverhampton, WV9 5AP, U.K

**Keywords:** Ecological survey, environmental DNA, great crested newt, real-time PCR, water samples

## Abstract

Current ecological surveys for great crested newts are time-consuming and expensive and can only be carried out within a short survey window. Additional survey methods which would facilitate the detection of rare or protected species such as the great crested newt (*Triturus cristatus*) would be extremely advantageous. Environmental DNA (eDNA) analysis has been utilized for the detection of great crested newts in Denmark. Here, the same methodology has been applied to water samples taken from UK ponds concurrently with conventional field surveying techniques. Our eDNA analysis exhibited an 84% success rate with a kappa coefficient of agreement between field and eDNA surveys of 0.86. One pond determined to be negative for great crested newt by field survey was positive by eDNA analysis, revealing the potential for improved detection rates using this methodology. Analysis of water samples collected in late summer indicates that eDNA analysis could be used to detect great crested newt after the optimal survey window for current field techniques had passed. Consequently, eDNA analysis could augment currently stipulated techniques for great crested newt surveying as a relatively quick and inexpensive tool for collecting great crested newt presence and distribution data within the UK instead of or prior to full field surveys.

## Introduction

Knowledge of species distribution is critical to ecological management and conservation biology. Effective management requires the detection of populations which can sometimes be at low densities and is usually based on visual detection and counting. Numerous publications now suggest that noninvasive sampling using environmental DNA (eDNA) for species-specific detection can be reliable and correlate well with conventional survey results (Ficetola et al. 2008; Jerde et al. 2011; Dejean et al. 2012; Foote et al. 2012; Thomsen et al. 2012b; Takahara et al. 2013) reviewed in (Rees et al. 2014). The use of eDNA analysis in monitoring and conservation of aquatic populations arose from the assessment of the diversity of macro-organisms in ancient sediments (Willerslev et al. 2003). Several different ancient and modern environments have been subject to this approach, for example terrestrial sediments, ice cores, and freshwater lakes and rivers (Hofreiter et al. 2003; Willerslev et al. 2003, 2007; Ficetola et al. 2008; Matisoo-Smith et al. 2008; Thomsen et al. 2012b). The first study on freshwater samples was carried out to track the presence of the invasive American bullfrog (*Rana catesbeiana*) considered to be one of the most harmful invasive species and responsible for the decline of native amphibians by direct predation, competition, diffusion of diseases, and complex biotic interactions (Blaustein and Kiesecker 2002; Kats and Ferrer 2003; Garner et al. 2006). Analyses showed that a multisampling approach allowed for the detection of the bullfrog even when it was present at low densities (Ficetola et al. 2008).

**Figure 1:**
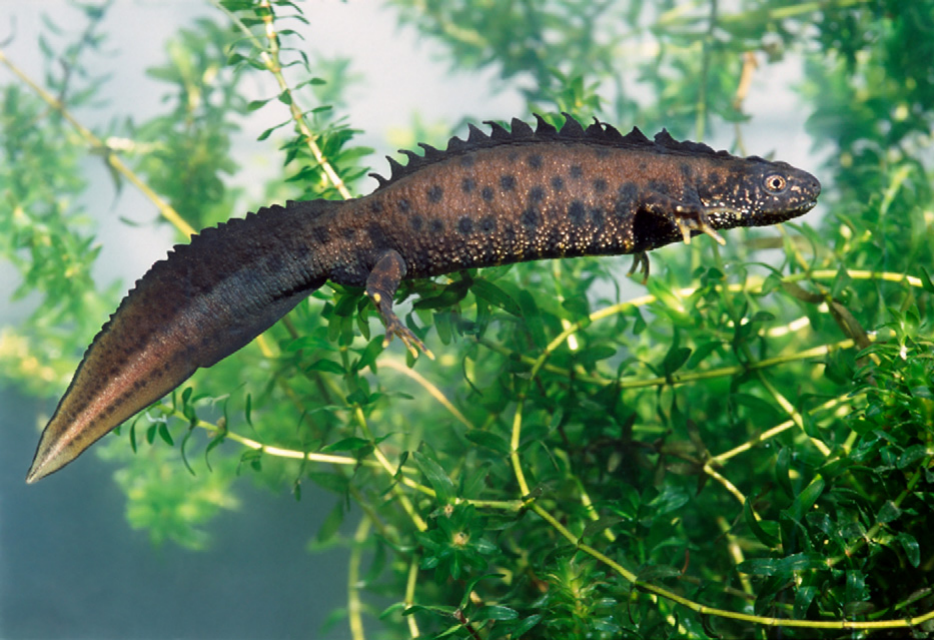
Male great crested newt (*Triturus cristatus*).

The total eDNA present includes DNA that originates from sloughed cellular material or that is excreted or secreted from animals occupying water bodies and similarly from animals that visit the environment, for example visiting the water body to drink. The presence of eDNA in water bodies demonstrates the presence or very recent presence of a particular species within that water body. DNA that is released into the environment is likely to be broken down and lost by the action of UV light and microbial activity over a period of around 2–4 weeks (Dejean et al. 2011; Thomsen et al. 2012a,b). eDNA target sequences are generally short amplicons (90–120 bp) based on abundant mitochondrial DNA sequences that are present in multiple copies per cell. Rapid diffusion of the eDNA from its source means that the presence of specific animal species can be detected within the water body and not just at its point of origin without the need for direct observations (Ficetola et al. 2008; Jerde et al. 2011; Dejean et al. 2012; Foote et al. 2012; Thomsen et al. 2012b; Takahara et al. 2013). This method is particularly useful for those species that are difficult to detect such as those that need trapping or require special surveying licences, as is the case for some endangered or protected species such as the great crested newt (*Triturus cristatus*) (Fig. 1).

In the UK, great crested newts (hereafter referred to as crested newt) are found across England, Scotland, and Wales but are largely confined to lowland areas (Beebee 1981). Crested newts require networks of permanent ponds and can move large distances between ponds or resting places with one study finding crested newts migrating up to 860 m to neighboring ponds (Kupfer and Kneitz 2000). Adults and juveniles normally live on land and hibernate between October and February (Langton et al. 2001). Their breeding season peaks in March to May, and during this time, crested newts are present in ponds and pools (Langton et al. 2001). Once hatched, the larvae live in these ponds until they develop into air-breathing juveniles after which they will begin to emerge from the ponds during August/September (Frazer 1983).

Environmental pressures threaten crested newts and other species due to the deterioration of aquatic and terrestrial habitats (Denoel et al. 2013). Crested newts are not only regionally threatened (Beebee 1997; Denoel and Ficetola 2008) but suffer from global decline (Denoel 2012) and as such all stages of the crested newt life cycle including their eggs are protected by UK and European law. This law means that proposals for land-use change which might affect the conservation of this species are obliged to survey for the presence of crested newts. In England, the ecological surveys are carried out under licence and conditions defined by Natural England. Survey methods consist of aquatic funnel traps, netting, torchlight, and egg counts to determine the presence of this species. Surveys consist of a minimum of four and up to six site visits between mid-March and mid-June, with at least two between mid-April and mid-May. This can therefore be both time-consuming and expensive for developers due to the number of site visits which may be necessary to establish reliable presence/absence data.

Here, we evaluate the potential of eDNA analysis to detect UK populations of crested newts as an alternative/additional methodology to field surveys using a real-time PCR primer and probe set for crested newt that has previously been described (Thomsen et al. 2012b). Water samples were collected from 38 ponds, 19 of which were known to contain crested newts and 19 were presumed to have an absence of crested newts, as determined by field survey. An artificial pond known to contain a crested newt population was used to demonstrate the persistence of detectable crested newt DNA within a defined water body and also to investigate the likely eDNA crested newt survey window. This is the first study to evaluate the crested newt eDNA monitoring technique on the UK crested newt population and its comparison to field survey data. We demonstrate that detection of UK crested newt was possible and that subtle modifications to the published PCR methodology could improve detection rates for this rare species.

## Materials and Methods

### Ethics statement

Crested newts are protected under the Wildlife and Countryside Act 1981 (as amended) and the Conservation of Habitats and Species Regulations 2010. Conventional field surveys for the GCN were carried out by ADAS ecologists under licence and specific conditions as set out by Natural England. Alongside these surveys, ecologists collected water samples from each pond. No vertebrate species were used directly; therefore, no IACUC or animal welfare protocol was required for water collection. GCN DNA was obtained from a deceased GCN under licence by Natural England (License Number 2014/SCI/0581 – Possession for sciences and education and conservation). All land was privately owned and was accessed with the permission of land owners for whom field surveys were being carried out.

### Field sampling and surveys

Field surveys were used to identify 19 crested newt-positive and 19 crested newt-negative ponds which were then subjected to eDNA analysis. Each pond was field surveyed on multiple visits using bottle trapping, torchlight surveys, and egg counting. A pond was counted as positive for crested newt if any of these three methods indicated the presence of crested newt (see Table 1).

**Table 1:** Summary of the crested newt survey and PCR status of the 38 ponds studied.

Pond number	Crested newt survey status	Standard PCR	Increased DNA volume PCR	Different visit	Additional analyses
1	Positive	1/12; 0/3	2/12; 1/3	–	
2	Positive	5/12; 2/3	8/12; 3/3	–	
3	Positive	1/12; 1/3	5/12; 3/3	–	
4	Positive	1/12; 1/3	3/12; 2/3	–	
5	Positive	1/12; 1/3	1/12; 1/3	–	14/72; 2/3
6	Positive	3/12; 2/3	7/12; 2/3	–	
7	Positive	0/12; 0/3	1/12; 1/3	–	12/72; 3/3
8	Positive	4/12; 1/3	5/12; 2/3	–	
9	Positive	1/12: 1/3	4/12; 3/3	–	
10	Positive	4/12; 2/3	9/12; 3/3	–	
11	Positive	0/12; 0/3	0/12; 0/3	1/12; 1/3^1^	2/72; 2/3
12	Positive	5/12; 2/3	9/12; 3/3	–	
13	Positive	0/12; 0/3	1/12; 1/3	–	4/72; 2/3
14	Positive	0/12; 0/3	0/12; 0/3	0/12; 0/3	
15	Positive	0/12; 0/3	0/12; 0/3	1/12; 1/3	5/72; 2/3
16	Positive	2/12; 1/3	1/12; 1/3	–	7/72; 2/3
17	Positive	0/12; 0/3	0/12; 0/3	–	
18	Positive	1/12; 1/3	5/12; 3/3	–	
19	Positive	0/12; 0/3	3/12; 2/3	–	
20	Negative	0/12; 0/3	0/12; 0/3	–	
21	Negative	0/12; 0/3	0/12; 0/3	–	
22	Negative	0/12; 0/3	0/12; 0/3	–	
23	Negative	0/12; 0/3	0/12; 0/3	–	
24	Negative	0/12; 0/3	0/12; 0/3	–	
25	Negative	0/12; 0/3	0/12; 0/3	–	
26	Negative	0/12; 0/3	0/12; 0/3	–	
27	Negative	0/12; 0/3	0/12; 0/3	–	
28	Negative	0/12; 0/3	0/12; 0/3	–	
29	Negative	0/12; 0/3	0/12; 0/3	–	
30	Negative	0/12; 0/3	1/12; 1/3	–	0/24
31	Negative	0/12; 0/3	0/12; 0/3	–	
32	Negative	0/12; 0/3	0/12; 0/3	–	
33	Negative	0/12; 0/3	0/12; 0/3	–	
34	Negative	0/12; 0/3	0/12; 0/3	–	
35	Negative	0/12; 0/3	0/12; 0/3	–	
36	Negative	0/12; 0/3	0/12; 0/3	–	
37	Negative	0/12; 0/3	0/12; 0/3	–	
38	Negative	5/12; 2/3	12/12; 3/3	–	
eDNA persistence *t* = 0 days	Positive	3/4; 1/1	4/4; 1/1	–	
eDNA persistence *t* = 6 days	Unknown	0/4; 0/1	0/4; 0/1	–	
eDNA persistence *t* = 15 days	Unknown	0/4; 0/1	0/4; 0/1	–	
August^2^	–	0/4; 0/1	1/4; 1/1	–	
September^3^	–	0/12; 0/3	0/12; 0/3	–	
October^4^	–	0/12; 0/3	0/12; 0/3	–	
November^5^	–	0/12; 0/3	0/12; 0/3	–	

Table showing the crested newt status of the 38 ponds by conventional survey, that is, bottle trapping, torchlight, and egg counts (crested newt survey status); standard PCR with the additional of 3 *μ*L DNA template; increased DNA volume PCR with the addition of 13 *μ*L DNA template; a different visit under increased DNA volume PCR conditions; and the results of additional analysis performed on ponds which had only 1/12 positive amplifications. PCR results are stated as the number of positive PCRs of the 12 PCR replicates, that is, based on 4 PCRs per sample and 3 water samples taken per pond, or of the 72 replicates for the additional analysis, that is, based on 24 PCRs and 3 water samples taken per pond. Hyphens in the “Different visit” columns illustrate those samples which were not tested by these methods.

1 Pond 11 was positive for samples from two additional visits as discussed in the text.

^2–5^August, September, October, and November correspond to the months during which water samples were collected from the artificial pond which was known to contain crested newt populations and was survey positive in April.

All surveys were conducted in accordance with the Great Crested Newt Mitigation Guidelines (English Nature, 2001). Survey methods consisted of aquatic funnel traps (bottle traps), netting, torchlight, and egg counts and took up to 3 h to perform. On an average-sized pond, this entailed preparation and setting up of bottle traps at a density of one per 2 m of shoreline shortly before sunset. Bottle traps were left over night for 12–14 h, but always less than 17 h before collection. Torching was carried out following sunset. The time taken to carry out egg counts was highly variable depending on the amount of vegetation present and on how quickly eggs were found. Once an egg was found, no further search was conducted.

Alongside the field surveys, 3 × 50 mL surface water samples were collected from each pond during at least one of the site visits and these were sent to the laboratory for crested newt eDNA analysis. Water samples were collected from 38 ponds (some on multiple occasions) during the 2012 or 2013 GCN survey seasons. To improve coverage of the water system and the chances of species detection, three water samples were taken from three different sites around the pond (Ficetola et al. 2008). 1 × 50 mL water samples for use as procedural controls were taken from bottle traps which had had crested newts positively identified within them. Additionally, for purposes of negative controls, 1 × 50 mL water samples were taken on 8 occasions from a well-characterized garden pond with no crested newt population. All samples were stored at −20°C immediately upon sample receipt.

### Artificial pond experiment

An artificial pond known to contain crested newt populations (10 years of observations) was used to provide water samples for an eDNA persistence study and the investigation of the survey window. This pond had dimensions 11 × 7 × 1.65 m (length × width × depth), and a nominal water body depth of 110 cm ± 10% underneath which was a 10- to 20-cm sediment layer comprised of base clay loam.

To investigate eDNA persistence, ecologists first confirmed the presence of the crested newt within the artificial pond by field observations prior to collecting a 10 L water sample. The water was removed to a crested newt-free location and kept under conditions of ambient temperature and light. 1 × 50 mL samples were taken from this 10 L water sample after 6 and 15 days to confirm the results of a previous crested newt DNA persistence study, that is, that eDNA degrades and becomes undetectable within 1–2 weeks (Thomsen et al. 2012b). All samples were stored at −20°C immediately upon sample receipt.

To investigate the crested newt eDNA survey window, up to 3 × 50 mL surface water samples were taken from the artificial pond each month between August and November 2013, that is, at time points between 16 and 29 weeks after crested newts were first observed. All samples were stored at −20°C immediately upon sample receipt.

### DNA extraction and real-time PCR

DNA extractions and PCR were performed in separate laboratories each with dedicated equipment and laboratory coats; PCR plates were set up within a UV sterilizable PCR cabinet. Water samples were defrosted at room temperature and 15 mL subsamples were added to 33 mL 100% ethanol and 1.5 mL 3 mol/L sodium acetate pH 5.2 and left at −20°C overnight to precipitate DNA. DNA was recovered by centrifugation (5000 g, 35 min, 6°C), the supernatant discarded, and the pellet air-dried. Resulting pellets were extracted using the DNeasy Blood and Tissue kit (Qiagen Valencia, California, USA) following the manufacturer's instructions and finally resuspended in 200 *μ*L of elution buffer. Extraction blanks consisting of tap water in place of pond water to test for cross-contamination were also included.

The primers used were TCCBL (5′-CGTAAACTACGGCTGACTAGTACGAA-3′) and TCCBR (5′-CCGATGTGTATGTAGATGCAAACA-3′) and probe TCCB.probe (5′-CATCCACGCTAACGGAGCCTCGC-3′) which amplify a 81-bp fragment of mitochondrial *cyt-b* from crested newts (Thomsen et al. 2012b). The primers and probe were tested on DNA extracted from a deceased crested newt under license from Natural England and all amplifications were positive. Primers and probe tested by both *in silico* and “wet laboratory” analysis did not detect DNA from *Triturus marmoratus* (marbled newt, a related but not UK native species), *Triturus carnifex* (Italian crested newt, invasive to UK), *Lissotriton vulgaris* (smooth newt, native to the UK), and *Lissotriton helveticus* (palmate newt, native to the UK), (Thomsen et al. 2012b; Biggs et al. 2014).

Real-time PCRs were performed on a Roche Lightcycler RC-480. PCRs were set up in a total volume of 30 *μ*L consisting of 3 *μ*L of extracted template DNA, 0.5 *μ*L of each primer (0.4 *μ*mol/L), 1 *μ*L of probe (0.1 *μ*mol/L), 15 *μ*L of TaqMan® Environmental Master Mix 2.0 (containing AmpliTaq GOLD DNA polymerase; Life Technologies Carlsbad, CA, USA), and 10 *μ*L ddH_2_O. The PCR included an initial incubation for 5 min at 50°C; then, a 10-min denaturation step at 95°C, followed by 55 cycles of denaturation at 95°C for 30 s and annealing at 60°C for 1 min. Each 96-well plate contained two positive controls and two negative controls: positive controls consisted of two separate crested newt trap water DNA extracts originally used as procedural controls; negative controls consisted of duplicates of the extraction blank and a PCR control with ddH_2_O in place of DNA template. The criteria for recording a PCR result was that all replicates of negative controls must be negative and that all replicates of positive controls must be positive. Each water sample was amplified four times, using the multitube approach (Taberlet et al. 1996) which gave 12 repeats per pond (3 water samples per pond). A pond was recorded as positive for crested newt if two or more of the 12 PCR replicates were positive. Where only one of 12 replicates was positive, samples from these ponds were re-extracted and retested. For the DNA persistence samples, 1 × 50 mL water sample was taken at each time point resulting in four PCR replicates per time point. The artificial pond samples were taken in triplicate (3 × 50 mL) during September to November resulting in the standard 12 repeats (per time point), and only 1 × 50 mL sample was taken in August resulting in four PCR replicates.

Additional PCRs were performed with an increased volume of template DNA of 13 *μ*L (4.33× the volume of DNA used in the standard PCR). PCRs were set up in a total volume of 30 *μ*L consisting of 13 *μ*L of extracted template DNA, 0.5 *μ*L of each primer (0.4 *μ*mol/L), 1 *μ*L of probe (0.1 *μ*mol/L), and 15 *μ*L of TaqMan® Environmental Master Mix 2.0 (containing AmpliTaq Gold DNA polymerase; Life Technologies). To investigate the possibility of nonspecific amplification, 8 individual water samples from a well-characterized garden pond with no crested newt population were extracted and amplified a total of 96 times (12 replicates each) using 13 *μ*L of template DNA per reaction. Additional replicates were set up and spiked with 3 *μ*L of crested newt DNA to investigate the potential for sample matrix effects, for example, the water chemistry, the presence of sediments, or water quality which could affect the potential of the sample to support DNA amplification.

### Statistical analyses

The correlation between the success rate and the time in transit was calculated using Pearson's correlation.

To measure the agreement between the two survey methods, that is, field survey and eDNA analysis, Cohen's kappa coefficient (Cohen 1960) was calculated as follows:


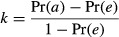


where Pr(*a*) is the relative agreement among rates, and Pr(*e*) is the hypothetical probability of chance agreement, using the observed data to calculate the probabilities of each method randomly giving a positive detection. If the methods are in complete agreement, then *κ* = 1. If there is no agreement other than what would be expected by chance, *κ* = 0.

Once all additional PCR analyses had been performed, the program PRESENCE version 6.4 [available from http://www.mbr-pwrc.usgs.gov/software/presence.html (Mackenzie et al. 2002)] was used for occupancy modeling of the data. A single-season model was used which assumes that species are never falsely detected at a site when absent, but that may or may not be detected when present; the detection of a species at an individual site is independent of the detection of the species at all other sites; and the probability of detecting the species across all sites is constant.

## Results

### Pond water experiments

The specificity of the primers and probe for crested newt was demonstrated under both standard PCR conditions and with an increased DNA volume. The negative control garden pond samples were subjected to PCR amplification to demonstrate the specificity of the primers and probe, this resulted in 0/96 positive reactions. To assess whether matrix effects or inhibitors present in these negative controls could have led to the possibility of false negatives, the negative control samples were spiked with crested newt DNA and subjected to PCR. All reactions were positive, showing that PCR amplification was possible under the conditions of the experiment. All extraction blanks were tested as negative. All positive and negative controls tested positive or negative as expected.

The success rate of the standard PCR was 63%, that is, 12/19 survey-positive ponds were PCR positive. To improve this success rate, the PCRs were repeated with 13 *μ*L DNA (a 4.33× increase in the volume of eDNA template). The success rate was increased to 79% (15/19) in survey-positive ponds.

To investigate whether the time of sampling can affect the success rate of the PCR, ponds 11, 14, and 15 (PCR negatives) were retested using water samples taken during a different survey visit. This was not possible for pond 17 as only water samples from one visit were available to us. At the increased DNA volume (13 *μ*L), this resulted in the detection of crested newt in ponds 11 and 15 (Table 1). This improved the eDNA PCR success rate to 89% of ponds tested, that is, 17/19 survey-positive ponds.

In the increased DNA volume PCR, 12 of the 19 ponds saw increases in the number of positive PCR replications compared to the standard PCR. One pond, pond 16, showed a decrease in the number of positive PCR amplifications upon increasing the volume of DNA in the amplification from 2/12 to 1/12; furthermore, the two amplifications were in a different water sample from the single amplification (samples three and one respectively). To confirm this result, the PCR was repeated six more times (72 PCR analyses), the numbers of positive amplifications varied from 0/12 to 2/12 with an average of 1.14/12 positives.

At the standard DNA volume, one of the ponds that was survey negative was PCR positive with 5/12 reactions being positive (Table 1). Increasing the volume of DNA in the reaction to 13 *μ*L increased this to 12/12 positive reactions.

All ponds with 1/12 positive amplifications were retested (see Table 1). Five of the seven ponds were confirmed as positive, giving a revised success rate of 84%. A sixth pond (Pond 11) showed positivity, but at a reduced level, making this a dubious positive, and a seventh pond (Pond 30) was negative.

### Artificial pond experiments

A limited set of DNA persistence experiments were performed, positive amplifications were found at time zero but not at 6 or 15 days after removal of water from the artificial pond.

To investigate the eDNA survey, window tests were carried out on an artificial pond sample taken in August. A standard PCR with 3 *μ*L of DNA template showed no amplification. However, when increased to 13 *μ*L of DNA, this artificial pond sample was PCR positive. Further samples were taken during September to November (three per time point) and analyzed at this increased DNA volume but were negative for crested newt DNA (see Table 1).

### Survey method performance

The success rate and time in transit (mean 1.58; range 0–4 days) were found to have a low negative correlation (−0.48, *P* < 0.05), illustrating that transit time between taking the water samples and freezing at −20°C did have an effect on the ability to detect the crested newt.

The average overall crested newt DNA amplification success in survey-positive ponds using increased DNA volume PCR and accounting for all reanalysis performed was 0.33 ± 0.24 (min: 0/12; max: 9/12, *N* = 19), and for survey-negative ponds, it was 0.06 ± 0.23 (min: 0/12; max: 12/12, *N* = 19).

Using the observed percentage agreement of the two methods of 0.89 (1 = 100%) and the probability of random agreement of 0.195, Cohen's kappa coefficient was calculated as 0.86 for survey-positive ponds versus their eDNA analysis results.

Site occupancy modeling was used to analyze the detection probability and quantify the effectiveness of the different approaches. Table 2 shows the parameter estimates for crested newts using field survey, standard PCR, increased volume PCR, or a combination of field survey and PCR. The use of increased volume PCR as compared to standard PCR increased the overall detection rate from 0.31 to 0.80 and also increased the occupancy estimate from 0.31 (95% CRI 0.23, 0.79) to 0.45 (95% CRI 0.30, 0.61). Increased volume PCR was found to have the highest overall detection rate at 0.80 with an occupancy rate of 0.45 (95% CRI 0.30, 0.61). Combining increased volume PCR with field survey increased the occupancy rate to 0.53 (95% CRI 0.37, 0.68) but did not further increase the detection rate as compared to increased volume PCR alone.

**Table 2:** Parameter estimates for field survey protocol, standard eDNA analysis, increased DNA volume eDNA analysis, or a combination of these methods using a single-season model *Ψ*(−), *p*(−),that is, assuming constant occupancy and detection.

Model	*N*	−2 Log likelihood	*Ψ* (95% CRI)	Est. *P* (95% CRI)	SE (*P*)
Field survey	2	97.51	0.50 (0.35, 0.66)	0.74 (0.58, 0.86)	0.072
Standard PCR	2	96.22	0.31 (0.23, 0.79)	0.31 (0.15, 0.54)	0.104
Increased volume PCR	2	102.47	0.45 (0.30, 0.61)	0.80 (0.66, 0.89)	0.060
Field survey plus standard PCR	2	195.57	0.51 (0.37, 0.69)	0.45 (0.37, 0.69)	0.051
Field survey plus increased volume PCR	2	182.52	0.53 (0.37, 0.68)	0.68 (0.59, 0.76)	0.046

Where *N* = number of parameters, *Ψ* = occupancy estimate, *P* = estimated detection rate. Sample size = 38 sites which for eDNA analysis was sampled at three points on one occasion, and for field survey sites were visited between 1 and 6 times.

## Discussion

The present study was carried out to evaluate the use of eDNA analysis to monitor the presence of the crested newt within ponds from the UK and to directly compare this to field survey data collected at the same time as the water samples. Crested newt eDNA was detected in water samples from 84% of the ponds where crested newt presence was observed by field survey methods such as bottle trapping, torchlight surveys, and egg counts. This resulted in an observed percentage agreement of 89% and a kappa coefficient of 0.86 which shows a good agreement between the results. When taken together, conventional ecological survey in combination with eDNA analysis using the increased DNA volume has led to improved crested newt detection rates, that is, 20 of 38 ponds rather than the 19 reported by field survey.

The success rate in survey-positive ponds (84%) was close to the 91% reported by Thomsen et al. (2012b); however, when applying the exact methods of Thomsen et al., our success rate was only 63%. It is possible that this lower success rate may be attributed to the time between sample collection and storage at −20°C which in our hands varied from samples being immediately frozen upon collection to up to 4 days before storage due to mail transit times. Given that eDNA becomes undetectable between 2 weeks or 1 month of the removal of the animals (Dejean et al. 2011; Thomsen et al. 2012b; Goldberg et al. 2013; Piaggio et al. 2013; Pilliod et al. 2014); and in the present study, we could not amplify eDNA from the eDNA persistence water sample taken after 6 days storage at ambient temperature and light, long transit times are not ideal. These results suggest that the stabilization of eDNA or its immediate extraction is of the upmost importance. In future, studies will need to adhere to a strict sampling regime where water samples are collected and immediately added to ethanol to minimize potential DNA degradation. DNA would then be recovered and stored at −20°C prior to PCR analysis.

Increasing the amount of template DNA from 3 to 13 *μ*L increased the success rate to 79% and is a simple step to improve detection rates. No studies reporting eDNA experiments have compared the PCR success rates with different amounts of template DNA; there is, however, a large variation in PCR design with reactions containing 0.5–10 *μ*L of template DNA. The use of large volumes of water (1–10 L) (Goldberg et al. 2011, 2013; Jerde et al. 2011, 2013; Minamoto et al. 2012; Olson et al. 2012; Thomsen et al. 2012b; Mahon et al. 2013; Pilliod et al. 2013b, 2014; Wilcox et al. 2013), normally used for eDNA species detection in rivers and streams due to the rapid dispersal of eDNA within river systems, might be tested to investigate its suitability for the detection of crested newt. These larger volumes of water are filtered to concentrate cellules or cellular remains rather than both cellular and extracellular DNAs as with methods involving DNA precipitation and as such may not be fully comparable; however, alternative methodologies warrant future investigation to determine those most suited to individual species.

Pond 16 may have exhibited PCR inhibiting matrix effects at the higher DNA volume although this seems unlikely as similar effects were not seen with all three individual samples. Indeed, matrix effects due to the addition of a greater volume of DNA were not observed in 14 of the 15 ponds that were subsequently positive as they all had an equal or greater number of positive PCR replicates than in the standard PCRs (Table 1). To confirm this result, further amplifications were performed (6 lots of 12 replicates) resulting in an average of 1/12 positive amplifications. Reactions containing negative pond water DNA spiked with crested newt DNA showed no inhibition, suggesting that matrix effects due to DNA concentration were unlikely to have caused the failures in amplification. Studies specifically investigating different water parameters, that is, different water chemistries, sediments, or quality which may demonstrate any correlation between water sample and eDNA detection rate, have not been reported, but such studies may help to explain possible reasons for PCR failures.

When additional samples from different visits were analyzed, it was shown that crested newt detection was possible in one of the three cases (Pond 15). This could suggest that multiple visits may be required to improve eDNA detection rates as per field surveys. A second sample (Pond 11) did show some levels of positivity but was classed as a dubious positive upon reanalysis. The result suggests that this represents a pond with an extremely low level of crested newt DNA and merely reflects the stochastic nature of PCR rather than there being zero DNA in the well. Alternatively, this could be due to the effects of long-term storage (∼1 year) of the sample prior to re-extraction and retesting, and any DNA present could have degraded to below the limit of PCR detection. To rule out inhibition effects, the spiking of samples with positive control DNA can be used to determine how appropriate a particular sample is to support DNA amplification. Only one group has tried to estimate the quality and quantity of template DNA prior to PCR amplification (Wilcox et al. 2013); several others have attempted to quantify the limits of detection and have developed standard curves for eDNA quantification (Takahara et al. 2012; Thomsen et al. 2012a,b; Goldberg et al. 2013; Pilliod et al. 2013a, 2014; Wilcox et al. 2013).

Species detection by eDNA and field work is likely to be imperfect and may lead to an underestimation of the distribution of a species especially in the case of rare or threatened species. Many species are difficult to detect during particular time periods or developmental stages, potentially biasing survey outcomes (Gotelli and Colwell 2001; Mackenzie et al. 2006). It is therefore likely that the data presented here represents an underestimation of the presence of the crested newt within the ponds tested. To achieve a higher level of coverage (especially for larger ponds), more samples may need to be taken. This could allow for the identification of sites within the pond where the crested newt would most likely be present, that is, at the edges of vegetation, thus improving the probability of detection. The ponds analyzed here were not measured; therefore, no correlation between size of pond and detection rates can be made, but it may be wise for future studies to do so.

Site occupancy models can be used to account for imperfect detection and have been used in amphibian surveys (Sewell et al. 2010) and were recently used by Schmidt et al. (Schmidt et al. 2013) to demonstrate their applicability to eDNA surveys. When applied to the data within this study, we found that the highest overall detection rate was achieved by increased volume PCR illustrating the utility of this methodology. Site occupancy estimates were no greater than the actual observed proportion but were increased though not significantly by combining field survey with increased volume PCR. This matches the observed increase in positive ponds from 19/38 to 20/38 when both techniques were combined.

One of the main issues with current field survey methodology for crested newt is the relatively short survey window which is optimal from mid-March to mid-June with a suboptimal window from July to October where only habitat searches and larvae netting can be used. Natural England currently stipulates that eDNA analysis can be between the 15 April and the 30 June. Where a positive result is obtained, field surveys could then be performed within the same survey season so long as two of the 4–6 visits can be performed between mid-May and mid-June. If this was not possible, then land development would be put on hold until the following survey season. A negative eDNA result, however, during this time could be used to support a development license application. Despite the very limited sample size, the detection of eDNA outside of the traditional survey window in a sample taken in August from an artificial pond is something which should be followed up by further research investigating the full “eDNA survey window” either side of the April–June window currently adopted.

In terms of sampling effort, analysis of eDNA can have considerable time and cost savings over traditional survey methods, especially when looking at the distribution of rare or threatened species. In a study of invasive Asian carp in Chicago, Illinois, it took 93 days of person effort to detect one silver carp by electrofishing at a site, whereas eDNA analysis required only 0.174 days person effort to achieve a positive detection (Jerde et al. 2011). In the case of crested newts, a field survey may take between 12 and 18 h of man time over several weeks of site visits and could cost several thousand pounds. eDNA analysis takes 20–30 min to collect the sample, and DNA extraction and PCR can be performed within a few hours at a cost of a few hundred pounds. We demonstrate here that eDNA analysis provides a relatively quick and inexpensive tool for collecting crested newt presence and distribution data.
